# Successful treatment of a free-moving abdominal mass with radiation therapy guided by cone-beam computed tomography: a case report

**DOI:** 10.1186/1752-1947-4-329

**Published:** 2010-10-19

**Authors:** Bouthaina Dabaja, Kelly J Perrin, Jorge E Romaguera, Patricia Horace, Christine F Wogan, Ferial Shihadeh, Mohammad R Salehpour

**Affiliations:** 1 Department of Radiation Oncology, The University of Texas MD Anderson Cancer Center, Houston, Texas 77030, USA; 2 Department of Lymphoma/Myeloma, The University of Texas MD Anderson Cancer Center, Houston, Texas 77030, USA; 3 Department of Radiation Physics, The University of Texas MD Anderson Cancer Center, Houston, Texas 77030, USA

## Abstract

**Introduction:**

Because tumors in the abdomen can change position, targeting these tumors for radiation therapy should be done with caution; use of daily image-guided radiation therapy is advised.

**Case presentation:**

We report the case of a 72-year-old Caucasian man with recurrent mantle cell lymphoma who was referred for palliative radiation therapy for an abdominopelvic tumor. Computed tomography was used to generate images for radiation treatment planning. Comparison of those planning images with a positron emission tomography/computed tomography scan ordered during the planning period revealed that the tumor had moved from one side of the abdomen to the other during the three-day interval between scans. To account for this unusual tumor movement, we obtained a second set of planning computed tomography scans and used a Varian cone-beam computed tomography scanner with on-board imaging capability to target the tumor before each daily treatment session, leading to successful treatment and complete resolution of the mass.

**Conclusion:**

Abdominal masses associated with the mesentery should be considered highly mobile; thus, radiation therapy for such masses should be used with the utmost caution. Modern radiation therapy techniques offer the ability to verify the tumor location in real time and shift the treatment ports accordingly over the course of treatment.

## Introduction

One of the most important challenges for the safe delivery of radiation therapy is the accurate application of three-dimensional conformal radiation therapy (3DCRT). The application of computed tomography (CT) in the 1970s to generate beam's-eye view images spurred the development of CT-based treatment simulation and planning for 3DCRT [1,2]. The benefit of conformal therapy lies in targeting the tumor area with smaller radiation fields while sparing the surrounding critical organs. But the benefit of better targeting came with the additional challenge of creating consistently reproducible means of positioning patients for multiple treatment sessions. Maintaining reproducibility among treatments involves multiple issues, including the devices used for patient immobilization and accounting for differences in set-up between sessions, changes in tumor size or volume between sessions, and the motion of internal organs during and between sessions. The International Commission on Radiation Units and Measurements (ICRU) addressed the issue of consistency in volume and dose specifications in radiation therapy in consecutive reports published between 1978 and 1999 [3-5]. These reports gave the radiation oncology community a consistent language and methodology for image-based, tumor volume-based treatment planning. Nevertheless, some patients present with tumors in locations that do not conform to known rules, and therefore treatments prescribed according to guidelines such as the ICRU reports can potentially miss the target and mistreat the patient. Here we describe the case of a patient who presented with an abdominopelvic lymphoma mass that could have been completely missed with conventionally planned treatment ports because of the extensive motion of the tumor within the abdomen.

## Case presentation

**Figure 1:**
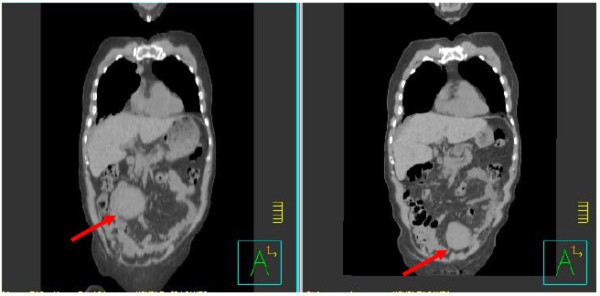
**Coronal treatment-planning computed tomography scans obtained five days apart showing the abdominal tumor in two distinctly different locations**.

**Figure 2:**
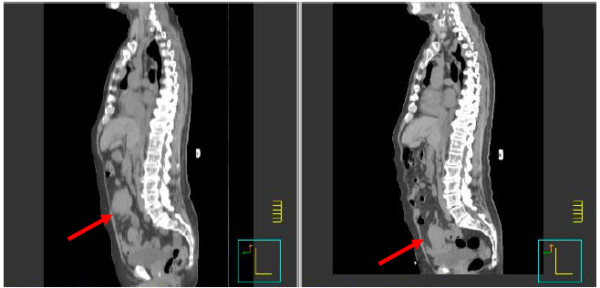
**Sagittal treatment-planning computed tomography scans obtained five days apart showing the abdominal tumor in two distinctly different locations**.

We present the case of a 72-year-old Caucasian man originally diagnosed in 2003 with stage IA mantle cell lymphoma, nodular pattern, involving the right parotid gland. At that time, he was treated with definitive radiation therapy to a total dose of 36 Gy, and the disease was in remission until 2006. He presented in July 2006 with shortness of breath and was found to have a right pleural effusion. Thoracocentesis confirmed the recurrence of mantle cell lymphoma. Disease restaging work-up revealed multicompartment lymphadenopathy in the neck, mediastinal, retrocrural, retroperitoneal and pelvic regions. Bone marrow was also involved. The patient was treated with a total of six cycles of rituximab, cyclophosphamide, vincristine, doxorubicin and dexamethasone (R-HyperCVAD) completed in January 2007. That treatment led to complete remission that lasted until October 2008, when the disease was found to have recurred in the left pleural space and retroperitoneum without bone marrow involvement. At that time, the patient was started on rituximab and lenalidomide but developed secondary and prolonged pancytopenia (white blood cell count, 1200 × 10^3^/μL [1200 × 10^9^/L]; neutrophils, 70%; hemoglobin, 7.6 g/dL [76 g/L]; platelets, 20 × 10^3^/μL [20 × 10^9^/L]) after the second cycle that precluded further chemotherapy or surgical resection. In April 2009, the patient was referred to radiation oncology to consider a palliative course of radiation to both the pleural-based and the retroperitoneal masses. The most urgent problems at that time were abdominal pain and early signs of bowel obstruction secondary to an abdominal mass. The 7.5 cm × 5.3 cm mass was located in the left midpelvic region within the small bowel mesentery anteriorly located beneath the abdominal wall. Coronal (Figure 1, left) and sagittal (Figure 2, left) CT scans showed that the mass extended from the lower part of vertebral body L5 to the upper part of vertebral body S2. Disease was evident in the mediastinum and right pleural area but was not causing any symptoms at that time, and the decision was made to administer palliative radiation to the abdominal mass.

Radiation treatment was simulated and planned based on CT scanning as follows. CT scans (5-mm slices) were obtained over the course of several days for planning purposes; the target volume was outlined on those scans, and a radiation therapy plan was generated by a Pinnacle treatment planning system (version 8.0, Philips Medical Systems, Madison, Wisconsin, USA). During the planning process, the medical oncologist ordered a positron emission tomography (PET)/CT scan. When the results of that scan became available three days later, the radiation oncologist noticed that the location of the tumor mass on the radiation planning CT scan was completely different than its location on the PET/CT scan. At that time, the patient was brought back to radiation oncology, another planning CT scan series was obtained, and the two sets of planning CT scans taken five days apart were compared. We found and confirmed that the tumor mass had moved in three dimensions, from the left side to the right side, from the lower pelvis to the above the pelvic rim, and from a mid anteroposterior location to a more anterior location, over those five days (Figures 1 and 2). We decided at that point to proceed with the treatment using a cone-beam CT device equipped with on-board imaging (Varian Medical Systems Inc., Palo Alto, California, USA). Cone-beam CT provides volumetric images in real time while the patient is immobilized in the treatment position immediately before each treatment session. We obtained cone-beam CT scans immediately before each daily treatment, which the treating radiation oncologist used to move the beam's-eye views (anteroposterior and posteroanterior) such that the tumor was contained within the radiation port. Because the extent of tumor motion ranged between 3 and 7 cm in all three dimensions and because we could not predict the direction or the extent of movement, we concluded that using cone-beam CT for daily verification of tumor position was the only way to effectively treat this mass.

The patient completed radiation therapy to a total dose of 36 Gy given in 18 treatment sessions. The volume of the mass decreased in from 213.9 cm^3 ^before the radiation therapy to 70.2 cm^3 ^at the completion of the radiation therapy.

**Figure 3:**
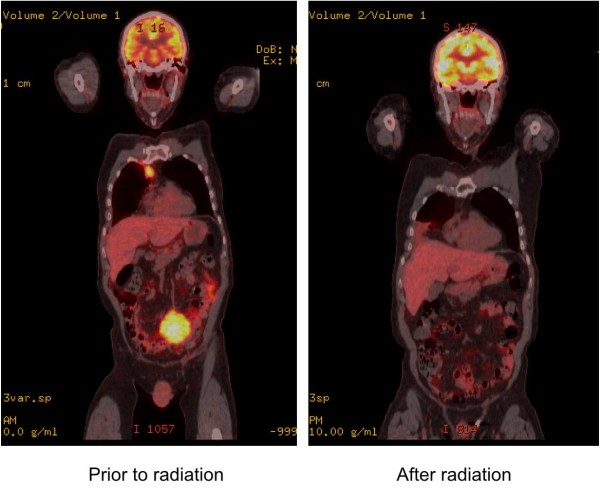
**Isodose lines drawn on transverse (left), sagittal (middle) and coronal (right) computed tomography (CT) scans illustrating changes in tumor location on the daily cone-beam CT images**.

**Figure 4:**
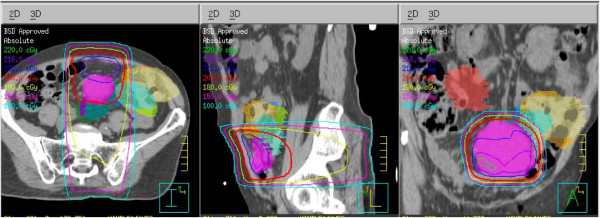
**Dose-volume histogram illustrating the doses that would have been delivered if cone-beam computed tomography had not been used**.

To assess the potential differences in dose distribution between the originally planned treatment and the treatment actually delivered with the use of cone-beam CT, we fused the original planning CT scans with the cone-beam CT scans obtained on selected treatment days and contoured the tumor to illustrate the shift in its location from day to day (Figure 3). We also generated dose-volume histograms for each daily tumor location assessment to show the doses that would have been received if the original planning fields had been applied without the use of cone-beam CT (Figure 4). That analysis shows that up to 80% of the tumor volume would have been missed in several instances.

**Figure 5:**
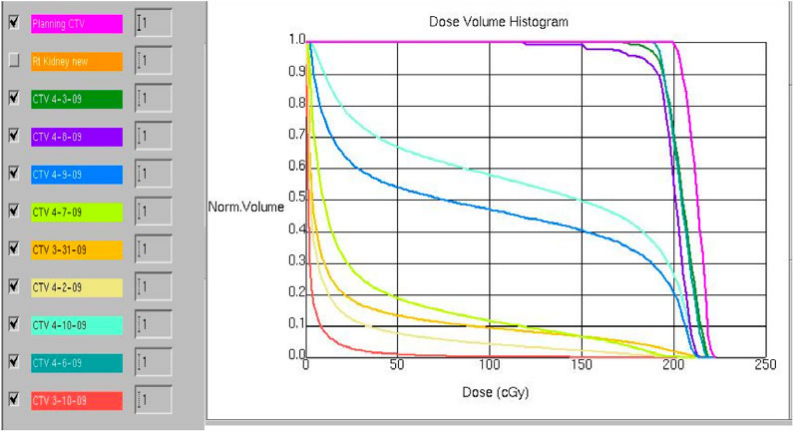
**Positron emission tomography/computed tomography images obtained before (left) and after (right) an 18-session course of radiation therapy**.

Side effects of the radiation treatment included diarrhea and fatigue. Because the mass never intercepted either kidney, no radiation was accidentally delivered to the kidneys. The patient returned for follow-up four weeks after completion of treatment, at which time a second PET/CT scan showed complete resolution of the treated mass (Figure 5) and no other masses in the abdomen.

## Discussion

Accounting for internal organ motion is a major problem in treating abdominal tumors with radiation therapy. Tumors attached to the mesentery can move significantly more than tumors located in the retroperitoneal region. Before the era of 3DCRT, the abdominal mass in this patient would have been missed in the course of daily treatments. Our use of cone-beam CT with on-board imaging capability was extremely useful in this case and allowed us to successfully treat this patient. Cone-beam CT was originally explored by Simpson *et al. *[6] as a way of generating single-slice tomograms with one gantry rotation of the linear particle accelerator (LINAC). Currently, several solutions involving CT image acquisition have been introduced into routine clinical use [7-9]. The concept of cone-based CT is based on integrating a kilovoltage (kV) x-ray source and a large-area flat panel detector on a standard LINAC to allow simultaneous fluoroscopy, radiography and volumetric kV cone-beam CT imaging. A volumetric CT image is reconstructed from data collected during a single gantry rotation.

The uncertainty of the dose distribution that would have been received by the tumor if conventional radiation planning techniques had been used is the core message of this report. We showed that the tumor would have been almost completely missed in several instances. Moreover, the cone-beam approach also allowed us to account for decreases in tumor size as well as position over the course of treatment, so the difference in planned dose distribution would have been even greater if daily changes in the tumor volume had not been accounted for. In light of the variable and unpredictable daily movement of the mesenteric mass, the tumor mass would definitely have received inadequate coverage and some days would have been completely missed by the radiation fields. We conclude from this experience that image-guided radiation therapy is both valid and useful for tracking the motion of highly mobile abdominal masses.

## Conclusion

This report is intended as a cautionary note to the radiation oncology community to use care when treating mesenteric-based masses in the abdomen because such masses can move substantial distances and can easily be missed if treatment is planned according to the current 3DCRT guidelines.

## Abbreviations

CT: computed tomography; kV: kilovoltage; PET: positron emission tomography; 3DCRT: three-dimensional conformal radiotherapy.

## Competing interests

The authors declare that they have no competing interests.

## Authors' contributions

BD analyzed and interpreted the patient data regarding the radiation treatment. MRS analyzed the technical data, particularly use of the cone-beam CT. PH helped obtain consent and provided patient care. KJP generated comparative plans. JER was the medical oncologist. CFW drafted the manuscript and revised it for intellectual content. All authors read and approved the final version of the manuscript.

## Consent

Written informed consent was obtained from the patient for publication of this case report and accompanying images. A copy of the written consent is available for review by the Editor-in-Chief of this journal.
